# Factorial Optimization of Secondary Annealing Parameters for Enhanced Magnetic Performance in M4 Grain-Oriented Electrical Steel Toroidal Cores

**DOI:** 10.3390/ma19112203

**Published:** 2026-05-23

**Authors:** Alma Lilia Moreno-Ríos, Luis Adrián Zúñiga-Avilés, José Martín Herrera-Ramírez, Caleb Carreño-Gallardo

**Affiliations:** 1 Posgrado CIATEQ AC, Centro de Tecnología Avanzada, Lerma de Villada 52004, Estado de Mexico, Mexico; alma.lilia.moreno.rios@gmail.com; 2 Facultad de Medicina, Universidad Autónoma del Estado de México, Av. Paseo Tollocan, Moderna de la Cruz, Toluca 50180, Estado de Mexico, Mexico; 3 Centro de Investigación en Materiales Avanzados, S.C. (CIMAV), Av. Miguel de Cervantes #120, Complejo Industrial Chihuahua, Chihuahua 31136, Chihuahua, Mexico; caleb.carreno@cimav.edu.mx

**Keywords:** grain-oriented electrical steel (M4), secondary annealing, transformer core losses, Goss texture, insulating coating, factorial design

## Abstract

Grain-oriented (GO) silicon steel cores in low-voltage current transformers suffer magnetic degradation from residual stress and increased dislocation density during slitting and winding. This study addresses the gap in systematic optimization of secondary annealing on assembled toroidal cores using a 3^2^ full-factorial design varying temperature (650, 850, 1050 °C) and holding time (60, 90, 120 min) on M4 grade cores. Results showed temperature is the dominant factor, while holding time exhibits a synergistic non-linear effect. The optimal condition (850 °C, 90 min) reduced specific losses from 0.85 W/kg to 0.43 W/kg (49% reduction). Mechanistic analysis confirmed this improvement is driven by complete primary recrystallization (equiaxed grains ~50–60 µm), dislocation annihilation (~10 HV hardness reduction), and reinforcement of the Goss texture ({110} <001>). SEM, EDS, and ICP-OES demonstrated that the Carlite coating remained dimensionally (1.67–1.83 µm) and chemically stable, with beneficial decarburization. Temperatures above 850 °C caused magnetic deterioration due to excessive grain growth. These results provide a validated, industrial framework for recovering magnetic efficiency in wound toroidal cores without compromising coating integrity.

## 1. Introduction

Power loss models for magnetic cores have been comprehensively classified and a theoretical framework established for understanding how annealing-induced microstructural modifications reduce hysteresis and Eddy current losses [1]. In this context, Grain-Oriented Electrical Steel (GOES) stands as one of the most critical soft magnetic materials for power distribution and measurement transformers, owing to its high magnetic permeability along the rolling direction and its low specific core losses. These functional properties arise from the strong Goss crystallographic texture ({110} <001>) [2], which aligns the easy magnetization axis with the rolling direction, and from the high silicon content (~3 wt.%) that raises electrical resistivity and thereby suppresses Eddy current losses [3].

The manufacturing of GOES involves a precisely engineered sequence of thermomechanical processing steps, among which secondary recrystallization annealing plays a decisive role in promoting abnormal grain growth of Goss-oriented grains and consolidating the final crystallographic texture. The thermodynamic and kinetic parameters governing this stage—including heating rate, applied pressure, and processing atmosphere—must be rigorously controlled, as they directly determine the microstructural evolution and the resulting magnetic performance of the material [4]. The global drive toward higher energy efficiency in electrical grids has renewed and intensified interest in further reducing core losses, both in conventional grain-oriented steel and in emerging soft-magnetic materials such as amorphous and nanocrystalline alloys [5].

The production of GOES involves a carefully engineered sequence of hot rolling, cold rolling, decarburization annealing, and high-temperature secondary recrystallization annealing, during which abnormal grain growth of Goss-oriented grains is promoted through inhibitor-dissolution mechanisms [6]. The intermediate annealing stage plays a critical role in governing primary recrystallization kinetics, grain boundary mobility, and final texture sharpness, as demonstrated for niobium-containing grade [7].

Precise control of annealing parameters temperature, soaking time, heating rate, and atmosphere is therefore essential for optimal mechanical and magnetic performance [8,9,10].

During annealing, several metallurgical phenomena occur, including recovery, recrystallization, and grain growth. Recrystallization leads to the formation of new, strain-free grains that replace the deformed microstructure generated during cold working, resulting in reduced hardness and increased ductility. Subsequently, grain growth reduces grain boundary density, which is particularly beneficial in magnetic materials, as it minimizes energy losses associated with domain wall motion [11].

In GOES, the final high-temperature annealing stage often referred to as secondary recrystallization annealing is essential for the development of the Goss texture ({110} <001>) [12]. This crystallographic orientation enables superior magnetic properties by aligning the easy magnetization direction with the rolling direction. The process is typically conducted under controlled atmosphere conditions at elevated temperatures, promoting abnormal grain growth of favorably oriented grains [13].

Therefore, precise control of annealing parameters such as temperature, soaking time, and heating-cooling rates is crucial for optimizing the microstructural evolution and magnetic performance of GOES. In this context, the present study aims to analyze the effect of annealing on the microstructure and its correlation with the functional properties of GOES sheets [14].

The development of the Goss texture during primary manufacturing of GOES is governed by a tightly coupled sequence of thermomechanical steps. As demonstrated by Fu and Fan [15], the microstructure and texture of the finished product can be traced back to the surface and quarter-thickness layers of the hot-rolled plate, where Goss-oriented grains originate and subsequently evolve through normalization, cold rolling, decarburization annealing, and high-temperature secondary recrystallization annealing. This continuous inheritance and transformation of crystallographic texture underscores that any post-fabrication thermal treatment capable of modifying the recrystallized grain structure—as in the secondary annealing applied to assembled toroidal cores in the present study—directly interacts with a texture state that is the cumulative result of the entire primary processing chain. After primary manufacturing, toroidal cores for current transformer applications are produced by slitting coils into narrow strips and winding them under tension. These mechanical operations introduce residual stresses, increased dislocation density, and lattice distortions that severely degrade magnetic domain wall mobility and increase both hysteresis and Eddy current losses [16]. The sensitivity of magnetic permeability to mechanical strain in highly grain-oriented electrical steels has been described through a domain-wall energy framework that captures the two-dimensional strain dependence of relative permeability, experimentally validated using a magnetic yoke system [17]. Analogous degradation has been documented in non-oriented electrical steel subjected to bending [18] and to punching and cutting operations [19], where annealing in the range 750–850 °C is commonly applied for stress relief.

Stress-relief annealing (SRA) has been extensively studied for flat laminations and Epstein strips of both grain-oriented and non-grain-oriented electrical steels. For grain-oriented grades, Cheng et al. [20], demonstrated that annealing at 850 °C for durations up to 8 h increases the average grain size from 42.3 µm to 68.2 µm in the thermally affected zone of wound-core transformers, confirming that residual stress elimination is the primary restoration mechanism for magnetic properties. Toktaş and Çelik [21] showed that precise thermal parameter optimization is indispensable for recovering magnetic flux density and reducing core losses in grain-oriented steels after mechanical deformation. Stasac et al. [22] characterized the direct correlation between grain spacing, hardness, and magnetic properties in GOES strips using an Epstein frame, establishing a quantitative link between microstructural modification and core loss behavior using methodology closely aligned with that of the present work. For non-grain-oriented steels, Hutchinson et al. [23] confirmed that residual dislocation substructures introduced by mechanical cutting are progressively annihilated during SRA at 800 °C, with recrystallization at cut edges identified as the governing restoration mechanism, producing significant power loss improvements. Alves et al. [24] further demonstrated that SRA temperature critically reduces magnetic anisotropy between rolling and transverse directions in shear-cut non-grain-oriented steel, with property recovery in the transverse direction identified as the dominant contributor to loss improvement.

The relationship between annealing temperature and magnetic core losses is inherently non-linear across silicon steel grades. Gao et al. [25] demonstrated in GOES that gradient thermal conditions during high-temperature annealing generate heterogeneous grain morphologies—including columnar and equiaxed grain populations—with distinct magnetic domain structures and different contributions to iron loss, confirming that the spatial homogeneity and rate of the thermal cycle are as critical as the peak temperature itself. This finding is directly relevant to the present study, where the slow heating rate and vacuum atmosphere employed during annealing of assembled toroidal cores were deliberately selected to minimize thermal gradients within the load. Complementary evidence from non-oriented silicon steels reinforces this principle: Chu et al. [26] showed that the relationship between annealing temperature and core loss exhibits a well-defined minimum at an intermediate temperature where the balance between recrystallization completion and grain growth onset is optimally controlled, with losses increasing again at higher temperatures due to excessive grain coarsening—a behavior entirely consistent with the results obtained in the present study for M4 grain-oriented toroidal cores and underscoring the industrial necessity of systematic parameter optimization rather than empirical selection of a single annealing condition. Collectively, these studies establish SRA as an effective strategy for magnetic property recovery, but remain predominantly restricted to individual laminations or Epstein strips processed before final assembly.

Despite this body of knowledge, critical gaps remain in the systematic study of SRA applied directly to fully assembled toroidal wound cores. First, most published studies focus on flat, unassembled laminations rather than on cores in their final geometric configuration, in which thermal gradients, mechanical constraints from the wound geometry, and the interaction between adjacent laminations may significantly alter the metallurgical response to annealing [27]. Second, the preservation of thin inorganic-organic insulating coatings—essential for inter-laminar electrical insulation—under SRA conditions has received limited attention specifically for assembled toroidal geometries; coating degradation during the thermal cycle would offset the magnetic gains achieved by stress relief [28]. Third, the systematic optimization of temperature and holding time through statistically rigorous experimental designs has not been applied to this configuration: existing work on toroidal cores either evaluates a single annealing condition or varies parameters without employing factorial methodologies capable of resolving interaction effects and non-linearities [29]. Fourth, the influence of vacuum atmosphere—as opposed to the nitrogen or argon atmospheres conventionally used in industrial SRA—on both the microstructural evolution and coating integrity of assembled toroidal cores has not been characterized. Finally, the relationship between annealing parameters, Goss texture sharpness, and core loss in toroidal wound-core geometries re-mains unresolved, particularly given recent evidence that texture fraction and grain size interact non-linearly to determine magnetic induction and iron losses [30,31].

The present study addresses these gaps by systematically optimizing a secondary annealing treatment applied directly to fully assembled M4 grain-oriented toroidal cores using a full-factorial 3^2^ experimental design. Unlike prior work conducted on flat laminations or individual strips, this approach evaluates the combined effect of annealing temperature (650, 850, and 1050 °C) and holding time (60, 90, and 120 min) on cores in their final wound geometry, under vacuum atmosphere, and on an industrial scale (400 cores per condition, 7200 cores in total). The primary response variable is specific magnetic core loss measured by the Epstein frame method according to IEC 60404-2 [32]. Secondary characterization—mechanical hardness, optical microstructure, X-ray diffraction (XRD) texture, scanning electron microscopy (SEM), energy-dispersive X-ray spectroscopy (EDS), and inductively coupled plasma optical emission spectrometry (ICP-OES) coating analysis—provided a mechanistic understanding of the observed magnetic improvements and supports the proposed industrial optimization framework [33].

## 2. Materials and Methods

### 2.1. Materials

Grain-oriented electrical steel grades M4, M5, and M6 share a predominant Goss crystallographic texture ({110} <001>) developed during secondary recrystallization processing, but differ in texture sharpness, sheet thickness, magnetic quality, and allowable specific core losses. M4 exhibits the lowest core losses and highest magnetic permeability, making it the preferred choice for high-efficiency transformer applications, whereas M5 offers intermediate magnetic performance and M6, while presenting higher core losses, represents the most economical option. Permissible core-loss classifications for these grades are defined by ASTM A876 [34], and magnetic testing and characterization procedures are established by IEC 60404-3 [35]. The maximum allowable specific core losses for each grade at 1.5 T and 1.7 T (50 Hz) are summarized in Table 1. The present study focuses exclusively on M4-grade GOES [23]. The selection of M4-grade GOES with Carlite coating is further supported by manufacturer specifications for Carlite-coated material (grades M-2 to M-6, 0.27 mm for M4), which confirm that Carlite coatings retain their insulating performance after stress-relief annealing (SRA), provided that appropriate annealing procedures are followed. Coating properties are preserved or even enhanced under neutral or slightly oxidizing atmospheres, and the LITE CARLITE variant is classified as C-5 insulation according to ASTM A976 [36], making it suitable for wound cores subjected to SRA under low to moderate volts-per-turn conditions [37]. From an economic perspective, comparative studies of M4 and high-permeability (H0) grades in dry-type transformer cores have demonstrated that, although M4 is a conventional grade, its cost-effectiveness is strongly dependent on achieving low no-load losses through optimized manufacturing processes, underscoring the industrial relevance of the annealing optimization pursued in the present work [38,39].

Commercial M4 GO silicon steel sheets (ThyssenKrupp Electrical Steel, Gelsenkirchen, Germany) with a nominal thickness of 0.27 ± 0.03 mm were used throughout this study [40]. The strong Goss texture ({110} <001>) confers high magnetic permeability along the rolling direction [40,41], with nominal specific core losses of 0.85 W/kg at 1.5 T and 1.25 W/kg at 1.7 T (50 Hz) in the as-received condition, as listed in Table 1. Toroidal cores with nominal dimensions of 112 × 92 × 50 mm (OD × ID × H) were fabricated from these sheets for low-voltage current transformer applications, with 400 cores processed per experimental condition. The sensitivity of magnetic permeability to mechanical strain in highly grain-oriented electrical steels has been described through a domain-wall energy framework that captures the two-dimensional strain dependence of relative permeability, experimentally validated using a magnetic yoke system [17]. This provides a theoretical basis for understanding how the winding-induced stresses introduced during toroidal core fabrication degrade domain wall mobility and, consequently, the magnetic performance of the assembled cores.

After annealing, each core was disassembled into Epstein strips of 30 mm × 280 mm for magnetic characterization according to IEC 60404-2 requirements. The bulk chemical composition corresponds to conventional GO grades, with a silicon content of approximately 3–4 wt.% Si [18], confirmed by ICP-OES at 3.76 wt.% in the as-received wound cores condition; low sulfur content and trace alloying elements contribute to increased electrical resistivity and reduced Eddy current losses [42]. Both surfaces were supplied with a commercial inorganic-organic insulating coating (Carlite), providing inter-laminar electrical insulation and thermal stability under stress-relief annealing conditions [43].

Toroidal cores for low-voltage current transformer applications were fabricated by slitting the sheets into narrow strips and winding them under tension according to industrial manufacturing specifications [44,45,46]. These forming operations introduced residual stresses, increased dislocation density, and localized lattice distortions that degraded the magnetic domain wall mobility and increased both hysteresis and Eddy current losses in the as-received wound cores condition [47,48]. This mechanical degradation constitutes the primary motivation for the secondary annealing treatment investigated in the present study.

### 2.2. Experimental Design of Secondary Annealing

A full-factorial 3^2^ design was employed to evaluate the effects of two independent factors, each studied at three levels, yielding nine unique experimental conditions. This design enables the analysis of both main effects and interaction effects on the response variable [49,50,51,52]. The use of full-factorial DOE for the optimization of heat treatment parameters in soft magnetic materials has been validated in recent studies; for instance, Kang and Lee [29] applied a factorial design comprising 96 unique parameter combinations to optimize compaction and heat treatment conditions in FeSi soft magnetic composites using toroidal specimens, evaluating compactability, density, and electromagnetic properties, thereby demonstrating the suitability of this experimental strategy for systematically mapping processing–property relationships in toroidal magnetic cores.

The two independent variables were: (1) maximum annealing temperature (T_max_) of 650, 850, and 1050 °C; and (2) holding time (t) of 60, 90, and 120 min. To ensure reproducibility and isolate the effects of these variables, all remaining process parameters were held constant throughout the study. All annealing treatments were carried out in a CAISA electromagnetic furnace C-12G DTT series 0H12 (WSE CAISA, Mexico City, Mexico), which provides superior thermal stability and temperature control compared with conventional gas-fired systems. Each experimental run involved a material batch of 450 kg of M4-grade GOES, determined by the furnace’s operational capacity. The total annealing cycle duration was standardized at 24 h, with a uniform initial temperature across all experimental trials.

Two key departures from conventional stress-relief annealing practice were implemented. First, whereas standard industrial procedures typically employ protective atmospheres (nitrogen or argon) to prevent surface oxidation, all treatments in this study were conducted under vacuum conditions [3 × 10^−2^ mbar]. Second, the annealing was applied directly to fully assembled wound toroidal cores rather than to individual laminations, eliminating the need for additional surface treatments or coatings during the thermal cycle. These process modifications constitute the distinguishing features of the experimental approach and are the subject of an ongoing patent application.

Each of the nine experimental conditions was performed in duplicate to improve statistical reliability, with replicate runs identified as Run 1R through Run 9R, resulting in 18 experimental runs in total. For each condition, 400 toroidal cores were processed—200 from batch A7177 and 200 from batch A7178—yielding 7200 cores processed across the full experimental campaign. The complete experimental matrix is presented in Table 2.

**Figure 1:**
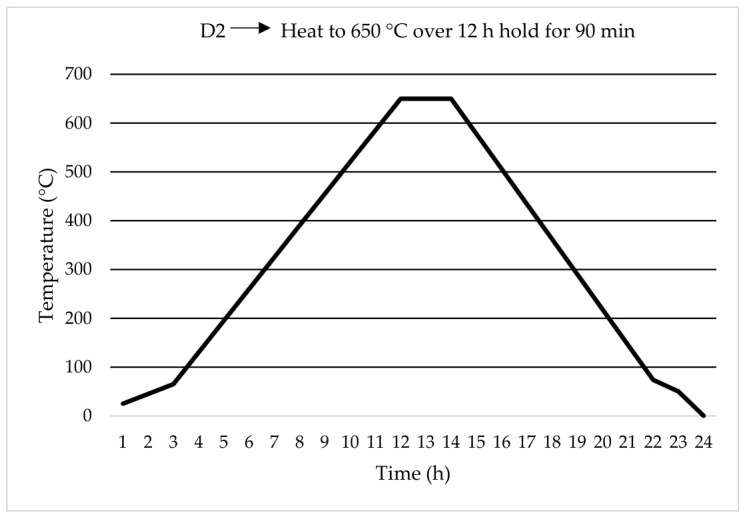
Thermal profile of the annealing cycle.

Temperature was monitored throughout each run using a calibrated thermocouple positioned near the load. The thermal profile consisted of a slow heating ramp to T_max_ over approximately 12 h, a holding stage at peak temperature for the specified duration, followed by furnace cooling to room temperature (Figure 1). The slow heating rate was selected to minimize thermal gradients within the load and prevent reintroduction of internal stresses during heating.

The selected temperature range enables the investigation of key microstructural mechanisms, including recovery, recrystallization, and grain growth, while the holding time governs the extent of microstructural evolution. In this experimental design, maximum temperature and soaking time are defined as the independent variables, allowing for the evaluation of both their main effects and interaction effects. The annealing process aims to reduce core losses, relieve residual stresses, and enhance magnetic permeability. Furthermore, the inclusion of replicates improves the statistical significance and reproducibility of the results. This systematic approach facilitates the identification of optimal processing conditions for improving the magnetic performance of electrical steel laminations.

### 2.3. Magnetic Characterization

**Figure 2:**
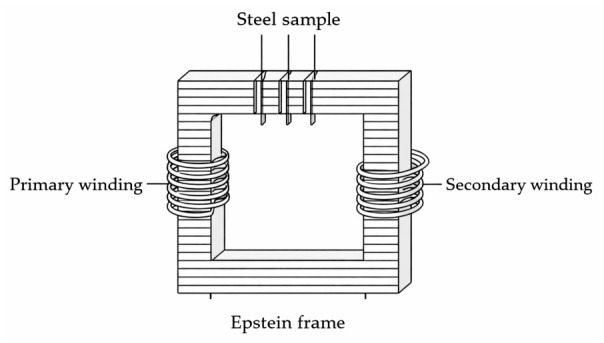
Epstein frame setup for magnetic loss measurement.

After annealing, each toroidal core was carefully disassembled into standardized strips of 30 mm × 280 mm for magnetic characterization using a TL2000 Epstein frame (Brockhaus Messtechnik GmbH & Co. KG, Lüdenscheid, Germany) according to IEC 60404-2 requirements (Figure 2). Approximately 42 strips were obtained from each core and assembled in the standard Epstein frame configuration. Measurements were performed at a frequency of 50 Hz and a peak magnetic flux density of 1.5 T. Total and specific core losses (W/kg) were recorded for each experimental condition and statistically analyzed to determine the main effects and interaction effects of temperature and holding time [53].

Table 3 summarizes the specific core loss values obtained across all 18 experimental runs of the 3^2^ factorial design. For each of the nine treatment conditions, measurements were performed on *n* = 800 cores in total (400 per replicate run), yielding paired datasets that enable direct assessment of inter-run reproducibility. Among all conditions, Run 5 (850 °C, 90 min) returned the lowest mean specific core loss of 0.43 W/kg, with a standard deviation of σ = 0.0072 W/kg and a coefficient of variation of CV = 1.67%, indicating high process stability under this condition. The negligible discrepancy between paired runs (e.g., Run 5 vs. Run 5R: Δ = 0.00 W/kg) confirms the thermal stability and reproducibility of the annealing cycle across independently processed batches A7177 and A7178. Equivalent agreement was observed across all nine treatment conditions, validating the statistical reliability of the full experimental dataset and supporting the subsequent factorial analysis of main effects and interaction effects.

The low data dispersion observed among the replicate runs confirms the high reproducibility and stability of the annealing process. Overall, the optimal annealing condition corresponds to an intermediate thermal regime that maximizes magnetic efficiency through adequate recrystallization and controlled grain growth.

### 2.4. Mechanical Characterization

Surface hardness was measured using a Wilson Rockwell 574 hardness tester (Wilson Instruments, Binghamton, NY, USA) in the HR15T superficial scale, with five independent indentations performed per condition to ensure statistical representativeness. The resulting HR15T values were converted to Vickers hardness (HV) using standard conversion tables to facilitate comparison with published literature on silicon steels. Hardness measurements were employed as a complementary indirect characterization technique, given that dislocation density, residual stress state, and recrystallization fraction—all of which are modified by annealing—are known to correlate with measured hardness in cold-worked metallic systems. Since these microstructural features simultaneously govern magnetic domain wall mobility and pinning, the evolution of hardness across annealing conditions provides mechanistic evidence supporting the interpretation of the observed changes in specific core losses.

### 2.5. Microstructural and Texture Analysis

Microstructural analysis was conducted by optical microscopy on cross-sections of as-received wound cores and annealed samples using an Olympus GX71 inverted metallurgical microscope (Olympus, Hamburg, Germany) equipped with Pax-it! image analysis software (Pax-it version 7.3, Villa Park, IL, USA). Grain morphology and recrystallization progression were evaluated qualitatively, while quantitative metallographic analysis was conducted in accordance with the ASTM E112 [54] standard using the circular intercept method. For this purpose, six samples were prepared from the lateral region of the toroidal core. A circular grid of known perimeter was superimposed onto micrographs taken at 100× magnification, and the intercepts between the grain boundaries and the circumference were counted. The average grain size was calculated as the ratio between the circumference length and the number of intercepts, with a total of 330 grains measured across all prepared specimens to ensure statistical significance. This procedure yielded an average grain size of approximately 59 µm with a standard deviation between 5.0 and 5.8 µm for the optimally annealed condition (850 °C, 90 min), where the observed variability is consistent with the heterogeneous grain growth behavior characteristic of primary recrystallization in grain-oriented electrical steels subjected to secondary annealing.

Crystallographic texture and phase evolution were analyzed by X-ray diffraction (XRD) using a Panalytical XpertPRO diffractometer (Malvern Panalytical, Almelo, The Netherlands). The diffraction patterns were recorded over a 2θ range covering the main BCC iron reflections (110), (200), (211), and (220), and peak intensities were compared between as-received wound cores and annealed conditions to evaluate lattice recovery and Goss texture retention. XRD measurements were performed on specimens of 15 mm × 20 mm, a sample size consistent with small-scale characterization approaches employed in the literature to resolve texture-related effects in electrical steels at high spatial resolution. In this regard, Leuning et al. [55] demonstrated that fundamental microstructural features—including grain orientation and deformation structures—exert a measurable influence on magnetic behavior and can be resolved using specimen configurations of comparable dimensions, supporting the representativeness of the sample geometry adopted in the present study.

### 2.6. Coating and Substrate Characterization

The surface morphology of the Carlite insulating coating was examined by scanning electron microscopy (SEM) using a Hitachi SU3500 microscope (Hitachi High-Technologies Corporation, Tokyo, Japan). Elemental composition and mapping were determined by energy-dispersive X-ray spectroscopy (EDS) coupled to the microscope. The bulk chemical composition of the steel substrate was verified by Inductively Coupled Plasma Optical Emission Spectroscopy (ICP-OES) using a PerkinElmer Optima 8300 spectrometer (PerkinElmer Inc., Waltham, MA, USA). Due to the limitations of ICP-OES for light elements, the carbon content was determined separately by combustion elemental analysis using a LECO TruSpec Micro CHN elemental analyzer (LECO Corporation, St. Joseph, MI, USA). Coating thickness was measured from SEM cross-sections at multiple locations, yielding values between 1.67 and 1.83 µm.

## 3. Results and Discussion

### 3.1. Effect of Annealing Parameters on Magnetic Loss

The factorial analysis revealed that maximum temperature is the dominant factor governing magnetic losses, while holding time exerts a secondary but synergistic effect. At 650 °C, losses remained in the range 0.65–0.67 W/kg, representing only a modest improvement over the as-received wound cores value of 0.85 W/kg. At this temperature, recovery mechanisms including dislocation rearrangement and partial annihilation occur, but recrystallization remains limited. Consequently, residual lattice strain continues to impede magnetic domain wall motion, consistent with reports of incomplete property restoration in electrical steels annealed below the recrystallization threshold [56].

When the temperature increased to 850 °C, a pronounced reduction in losses was observed, reaching a minimum of 0.43 W/kg at a holding time of 90 min—an approximately 49% reduction compared to the nominal as-received wound cores value of 0.85 W/kg. At this condition, sufficient thermal activation energy promotes: (i) complete dislocation annihilation; (ii) full primary recrystallization producing strain-free equiaxed grains; (iii) elimination of residual internal stress; and (iv) improved domain wall mobility. These mechanisms are consistent with published results on grain-oriented steel, where annealing above 750–800 °C is required for full recovery of Goss texture and the associated low-loss properties [57]. The significance of this temperature range is further corroborated by You et al. [58], who analyzed heat treatment effects in soft magnetic materials using crystal recrystallization theory and grain evolution simulations over a temperature range of 800–920 °C. Those authors established and experimentally validated by SEM a temperature–grain size–magnetic energy correlation model, reporting variations of up to 22% in the initial magnetization range as a function of heat treatment temperature, which underscores the sensitivity of magnetic response to precise thermal control. Additionally, stress-relief annealing temperature has been shown to play a critical role in reducing magnetic anisotropy between the rolling and transverse directions in shear-cut non-grain-oriented electrical steel, where recovery and recrystallization near the cut edges are identified as the primary restoration mechanisms, with improvements in average core losses attributed mainly to property recovery in the transverse direction [24].

Further increasing the temperature to 1050 °C resulted in significant deterioration of magnetic performance (0.70–0.75 W/kg), despite the additional thermal energy available. This behavior is attributed to excessive grain growth and disruption of the optimized Goss domain configuration. Abnormal grain growth at elevated temperatures can introduce misoriented grains and increase localized magnetic anisotropy dispersion, counteracting the benefits of dislocation removal [59]. Holding time exhibited a non-linear effect at each temperature level; at 850 °C, the 90 min condition produced the lowest losses, while shorter (60 min) or longer (120 min) times resulted in higher losses, suggesting incomplete recrystallization and onset of uncontrolled grain growth, respectively [60]. The 49% improvement measured by the Epstein frame is corroborated by direct voltage induction measurements on assembled cores (from 0.0313 V to 0.0588 V after annealing), confirming the consistency of the result across two independent methods. The mechanistic interpretation of these results is reinforced by the work of Cheng et al. [20], who investigated the microstructure, crystallographic grain orientation, and magnetic performance of GOES subjected to stress-relief annealing by means of SEM and electron backscatter diffraction (EBSD). Their results demonstrated that annealing at 850 °C for durations of up to 8 h increased the average grain size from 42.3 µm to 68.2 µm in the thermally affected region, and confirmed that the primary effect of the heat treatment is the elimination of residual stresses, which restores magnetic properties and reduces no-load losses in three-dimensional wound-core transformers—an observation directly consistent with the magnetic recovery and grain size evolution reported in the present study.

**Figure 3:**
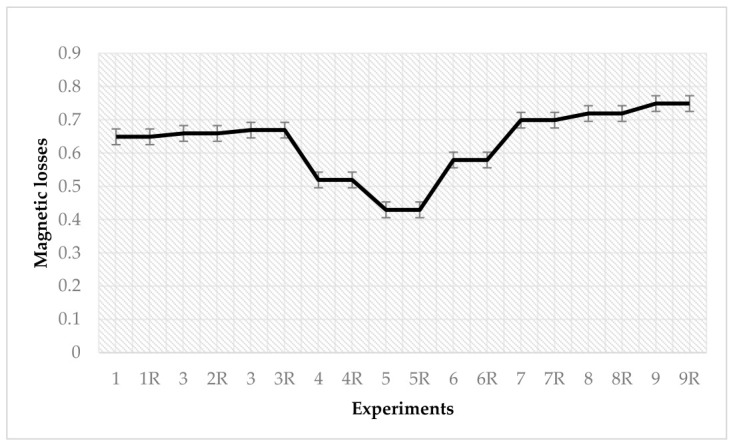
Specific magnetic core losses for all 18 experimental runs.

The results presented in Figure 3 demonstrate that an intermediate processing condition, specifically 850 °C for 90 min, provides the optimal balance between recrystallization and grain growth, leading to minimized core losses. Lower temperatures are insufficient to fully develop the desired microstructure, while excessive temperatures result in degradation of magnetic properties. Therefore, precise control of annealing parameters is essential to achieve enhanced magnetic efficiency and material performance. This conclusion is further corroborated by Paltanea et al. [61], who reported that the application of stress-relief annealing produces convergence and overlap of all magnetization curves in grain-oriented electrical steel strips subjected to mechanical cutting, confirming that annealing effectively eliminates cutting-induced residual stresses and fully restores the magnetic performance of the material—a behavior directly analogous to the property recovery observed in the present study following the winding-induced degradation of the toroidal cores.

### 3.2. Hardness and Stress Relief

**Figure 4:**
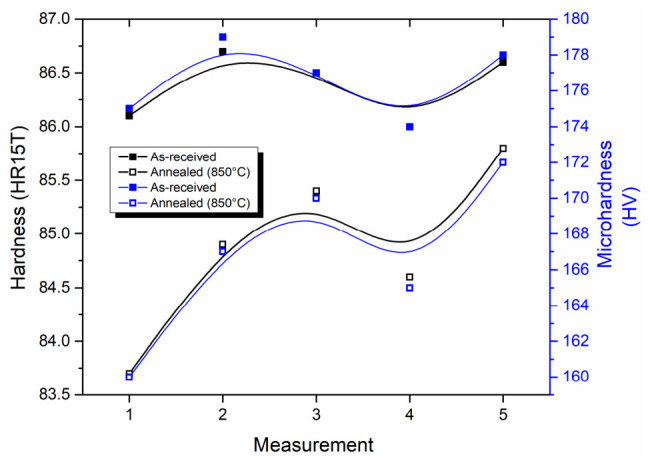
Evolution of surface hardness across five measurement points for the as-received wound cores and the optimally annealed condition (850 °C, 90 min): Rockwell HR15T superficial hardness (left axis) and converted Vickers hardness (HV, right axis). The black square markers indicate the discrete measurements for the as-received wound cores, while the blue square markers represent the optimally annealed condition; the corresponding solid lines serve as visual guides (interpolations) between the measurement points.

Secondary annealing at the optimal condition (850 °C, 90 min) produced a measurable reduction in surface hardness, from 176.6 HV (86.4 HR15T) in the as-received wound cores to 166.8 HV (84.9 HR15T) after treatment, representing a decrease of approximately 10 HV (~5.5%), as summarized in Table 4 and illustrated in Figure 4. This reduction is consistent with the progressive annihilation of geometrically necessary dislocations and the release of stored deformation energy accumulated during cold rolling and tension winding, both of which are characteristic signatures of recovery and primary recrystallization in silicon steels [62].

The as-received wound cores condition is characterized by strain hardening resulting from prior cold work, manifested as a high dislocation density, elevated stored deformation energy, and significant residual internal stresses—all of which act as pinning centers for magnetic domain walls. In contrast, the annealed condition at 850 °C presents a more stable and energetically favorable microstructure, wherein recovery and primary recrystallization have collectively reduced dislocation density and relieved residual stresses in a moderate and spatially homogeneous manner. This microstructural state is consistent with the functional requirements of grain-oriented silicon steel for electrical applications, where a low defect density is essential for minimizing hysteresis losses and maximizing domain wall mobility [63].

The magnitude of the hardness reduction is mechanistically significant: a ~10 HV decrease indicates sufficient thermal activation for dislocation annihilation and grain boundary migration without inducing the excessive softening associated with abnormal grain growth, which would compromise the dimensional and mechanical integrity of the assembled toroidal cores during subsequent handling and assembly operations. The correlation between this structural relaxation and the 49% reduction in specific core losses (from 0.85 to 0.43 W/kg) reflects a consistent mechanistic link between dislocation density reduction and magnetic performance recovery, in agreement with reported behavior for stress-relief annealing in grain-oriented electrical steels [38,39]. The degradation of magnetic properties resulting from mechanically induced residual stresses during core fabrication represents a well-documented challenge in the processing of electrical steels. Toktaş and Çelik [21] demonstrated that stress-relief annealing is indispensable for recovering magnetic flux density and reducing core losses in grain-oriented steels subjected to mechanical deformation, emphasizing that precise optimization of thermal processing parameters is critical to restore the magnetic integrity of the material—a finding that provides direct contemporary validation for the secondary annealing approach applied to toroidal core geometries in the present study. Complementary evidence is provided by Stasac et al. [22], who examined the effect of heat treatment on grain spacing, hardness, and magnetic properties in GOES strips characterized by means of an Epstein frame, with particular emphasis on the correlation between microstructural modifications and core loss behavior. The methodology and response variables employed in that study are closely aligned with those of the present work, enabling cross-validation of the hardness reduction and magnetic loss improvement results reported here.

The optimal heat treatment produced a moderate and homogeneous reduction in hardness as a result of recrystallization processes, leading to a significant decrease in dislocation density and residual internal stresses. In contrast, the untreated condition exhibits strain hardening due to prior cold work, characterized by a high density of dislocations and stored deformation energy. The annealed (850 °C) condition therefore presents a more stable and energetically favorable microstructure, consistent with the functional requirements of silicon steel for electrical applications, where reduced defect density enhances magnetic performance and minimizes hysteresis losses.

### 3.3. Microstructural Evolution and Texture

**Figure 5:**
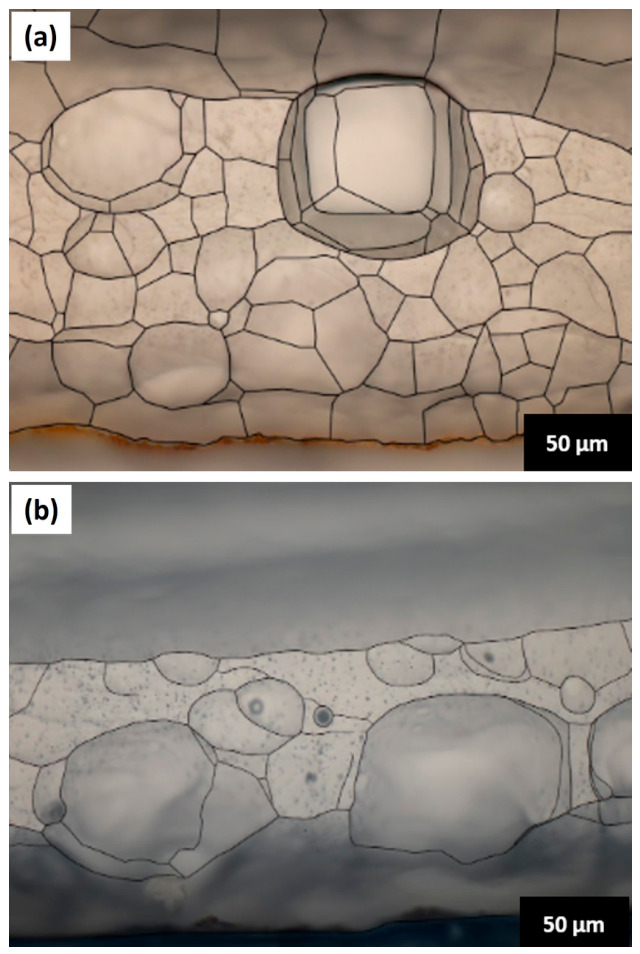
Optical micrographs of the annealed M4 steel (850 °C, 90 min): (**a**) lateral cross-section and (**b**) surface section showing the microstructure after the optimal annealing treatment.

Optical microscopy analysis (Figure 5) reveals a distinct microstructural transition from the initial state to the post-annealing condition. The as-wound samples exhibited elongated, mechanically deformed grains with high internal strain induced by the winding process. In contrast, the specimens subjected to secondary annealing at 850 °C showed a full transition to an equiaxed, strain-free recrystallized morphology. As outlined in the methodology, the quantitative analysis of 330 grains from the lateral region of the core resulted in an average grain size of approximately 59 µm, with a standard deviation (σ) ranging between 5.0 and 5.8 µm. These results indicate a relatively homogeneous microstructure, which is consistent with the expected stable magnetic performance for M4 grade steel. The transition from the elongated, deformation-induced grain morphology observed in the as-received wound cores condition to this equiaxed structure confirms that the secondary annealing at 850 °C for 90 min promoted complete primary recrystallization. This grain size falls within the optimal range for minimizing core losses by facilitating magnetic domain wall mobility. The presence of incipient larger grains (within the 70–100 µm range) in the lateral sections is consistent with localized preferential grain growth. This phenomenon is a critical precursor to the secondary recrystallization required for the stabilization of the Goss texture ({110} <001>), which is fundamental for enhancing magnetic permeability and reducing hysteresis losses in GOES toroidal cores. The transition from the elongated, deformation-induced grain morphology of the as-received wound cores condition to the equiaxed grain structure confirms advanced recrystallization at this annealing condition. This heterogeneous distribution is characteristic of grain growth following recrystallization in silicon steels, where abnormally large grains raise the mean value above that of the majority grain population [64]. The average grain size of ~59 µm falls within the range commonly associated with low core losses in M4 grade materials. These findings indicate a heterogeneous distribution associated with diffusion and grain growth processes. This behavior is characteristic of phenomena such as recrystallization and annealing, where the reduction in the material’s internal energy promotes grain coarsening. In comparison with the as-received condition, where finer grains and a higher density of crystalline defects typically predominate, the annealing heat treatment condition results in a more stable microstructure but with lower mechanical strength and higher ductility, in accordance with the Hall–Petch relationship, which establishes that an increase in grain size leads to a decrease in material strength.

The experimentally determined average grain size of ~59 µm (σ = 5.0–5.8 µm) reflects a heterogeneous microstructure characteristic of primary recrystallization in grain-oriented electrical steels, where the coexistence of equiaxed recrystallized grains with incipient larger grains in the 70–100 µm range drives an upward shift in the mean grain size distribution. This heterogeneity is consistent with the concurrent operation of nucleation and grain boundary migration during annealing and does not compromise the functional homogeneity required for stable magnetic performance in M4 grade cores.

**Figure 6:**
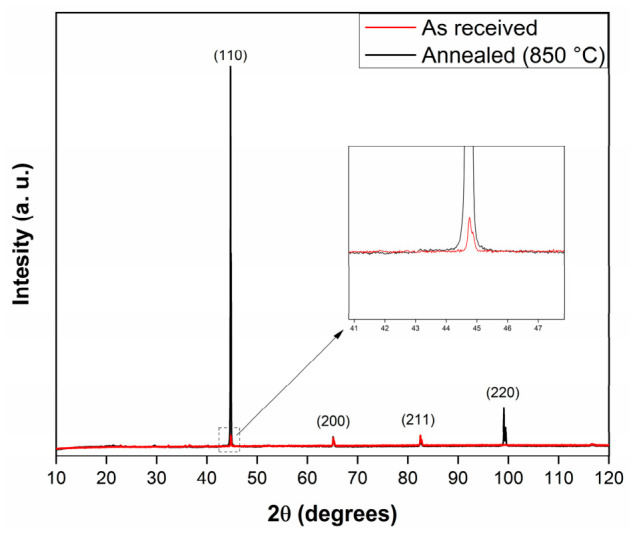
XRD patterns comparing the as-received wound cores and annealed conditions.

The XRD patterns in Figure 6 provide compelling crystallographic evidence of the microstructural changes induced by secondary annealing. In the as-received wound cores condition, the (110) BCC reflection shows reduced peak intensity and appreciable broadening, reflecting the high dislocation density and lattice strain introduced during slitting and winding operations. After annealing at 850 °C, a pronounced increase in (110) peak intensity is observed, accompanied by peak narrowing—quantified by a reduction in full width at half maximum (FWHM)—confirming dislocation annihilation, recovery of long-range crystalline order, and an increase in coherent diffraction domain size [65]. The reinforcement of the (110) reflection is particularly significant in the context of grain-oriented electrical steels, as this plane family corresponds to the Goss texture component ({110} <001>), whose recovery after mechanical degradation is directly responsible for the restoration of low-loss magnetic behavior along the rolling direction. The concurrent reappearance of the (200), (211), and (220) reflections with increased intensity further confirms the progression of primary recrystallization and the restoration of a well-ordered BCC ferritic matrix [66]. At 1050 °C, the broadening of diffraction peaks and reduction in peak sharpness are consistent with excessive grain growth and increased microstructural heterogeneity, which degrade the Goss texture sharpness and increase localized magnetic anisotropy dispersion [67].

The sensitivity of magnetic properties and iron losses to texture evolution under varying annealing conditions has been systematically demonstrated by Niu et al. [30], who showed that annealing tension modifies the η-fiber texture intensity ({001}), grain size, and iron losses in ultra-thin GOES in a non-linear manner, with both magnetic induction and core losses responding sensitively to changes in texture fraction and grain size—a finding that reinforces the critical role of texture control in determining the magnetic outcome of the annealing cycle. The dependence of Goss texture stability on annealing temperature is further substantiated by Liang et al. [31], who analyzed secondary recrystallization in 6.5% Si grain-oriented steel by EBSD and demonstrated that high-energy grain boundaries (20–45°) associated with Goss-oriented grains become progressively more frequent with increasing annealing temperature, establishing that the precise development of Goss-oriented grains during high-temperature annealing remains the central metallurgical challenge for achieving favorable magnetic properties in GOES.

### 3.4. Insulating Coating Stability

**Figure 7:**
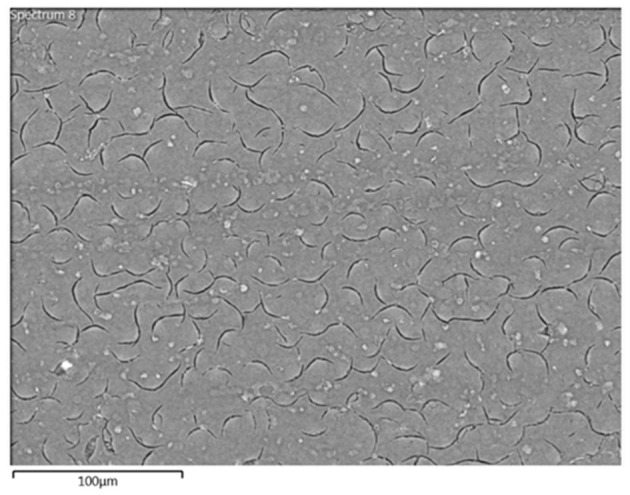
SEM surface morphology of the Carlite insulating coating after annealing.

SEM surface analysis in Figure 7 revealed a homogeneous coating morphology after annealing at 850 °C, with no evidence of delamination, cracking, blistering, or macroscopic degradation. Small bright-contrast particles dispersed across the surface are consistent with second-phase precipitates—likely oxides or sulfides—commonly observed in grain-oriented silicon steels. The lack of coalescence among these particles confirms that the annealing thermal cycle did not promote significant precipitate coarsening, indicating that diffusion-driven processes remained within a range that favors surface stabilization without inducing microstructural heterogeneity detrimental to coating integrity [68,69].

**Figure 8:**
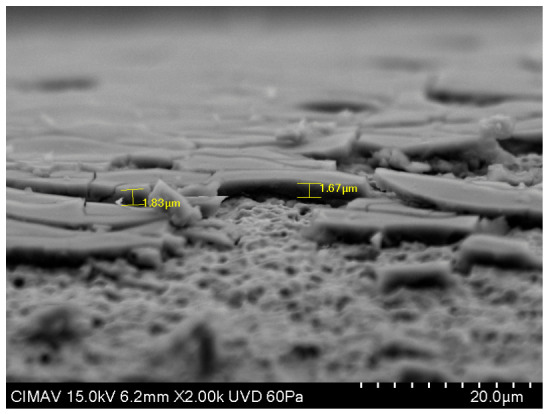
Secondary electron SEM cross-section showing the Carlite insulating coating thickness and the M4 electrical steel substrate.

The SEM cross-sectional analysis in Figure 8 confirmed that the Carlite coating thickness remained within 1.67–1.83 µm (average ~ 1.75 µm) after the optimized annealing cycle, with a total variation below 0.2 µm. This dimensional stability is critical for transformer core performance, as any reduction in coating continuity or inter-laminar insulation effectiveness would introduce additional Eddy current paths between adjacent laminations, partially or fully offsetting the hysteresis loss improvements achieved by stress relief [70,71]. The functional relevance of coating integrity extends beyond electrical insulation: Cheng et al. [28], demonstrated, through optical microscopy and EBSD analysis of high-permeability GOES with thicknesses of 0.18–0.27 mm, that coating-induced surface stress, magnetic domain refinement, and reduced domain wall velocity collectively contribute to the mitigation of anomalous losses. Their study further showed that core losses are highly sensitive to grain morphology—particularly to the presence of columnar grains under DC bias excitation—reinforcing the importance of preserving both coating integrity and the recrystallized grain structure achieved under the optimized annealing condition.

EDS and ICP-OES analyses confirmed negligible compositional variation in the major alloying elements between the as-received and optimally annealed conditions (Table 5), demonstrating that the thermal cycle does not alter the bulk chemical stability of the M4 substrate. Silicon content remained essentially constant at ~3.8 wt.%, consistent with the preservation of intrinsic electrical resistivity and the associated suppression of Eddy current losses. A particularly significant result was the reduction of carbon from 0.013 wt.% in the as-received wound cores to below the detection limit after annealing at 850 °C. This decarburization, facilitated by the vacuum atmosphere employed during the thermal cycle, is beneficial for core loss performance, as interstitial carbon atoms constitute effective domain wall pinning centers whose elimination contributes directly to hysteresis loss reduction [68,69]. The progressive elimination of deformation-induced dislocation substructures during stress-relief annealing, and its direct consequence on magnetic loss recovery, was independently corroborated by Hutchinson et al. [23], who compared SRA at 800 °C under nitrogen atmosphere on Epstein strip samples of non-grain-oriented electrical steel prepared by mechanical shearing and water-jet cutting. Their microstructural analysis confirmed that residual dislocation substructures introduced by mechanical cutting are progressively annihilated during annealing, with recrystallization at the cut edges identified as the governing restoration mechanism, leading to significant power loss improvements in low-aluminum steel grades—a mechanistic sequence directly analogous to the dislocation annihilation and core loss recovery documented in the present study for winding-induced deformation.

Taken together, the SEM, EDS, and ICP-OES characterization demonstrates that the Carlite insulating coating retained full morphological, dimensional, and chemical stability under the optimized annealing condition (850 °C, 90 min), confirming the compatibility of this processing window with the operational requirements of the insulation system. At higher temperatures (1050 °C), the observed magnetic deterioration may be partially attributable to excessive grain growth and incipient thermal instability of the insulating layer; however, direct experimental evaluation of coating integrity at this condition was not performed in the present study and constitutes a relevant subject for future investigation.

The comparative analysis of the chemical composition between the annealed (850 °C) and the as-received samples indicates no significant variations in the major alloying elements, confirming that heat treatment does not alter the bulk chemical composition but primarily affects the microstructure. The silicon content remains essentially constant (~3.8 wt.%), ensuring stable intrinsic magnetic properties. Minor differences observed in elements such as P and S are within experimental variability and may be associated with segregation or measurement uncertainty. Notably, carbon is undetectable after heat treatment, whereas it is present in the as-received wound cores condition, suggesting decarburization during annealing. This reduction in carbon is critical for improving magnetic performance by minimizing hysteresis losses. Overall, the results demonstrate that the heat treatment process mainly promotes microstructural evolution while maintaining chemical stability, with localized compositional changes such as carbon reduction playing a key role in enhancing material performance.

**Figure 9:**
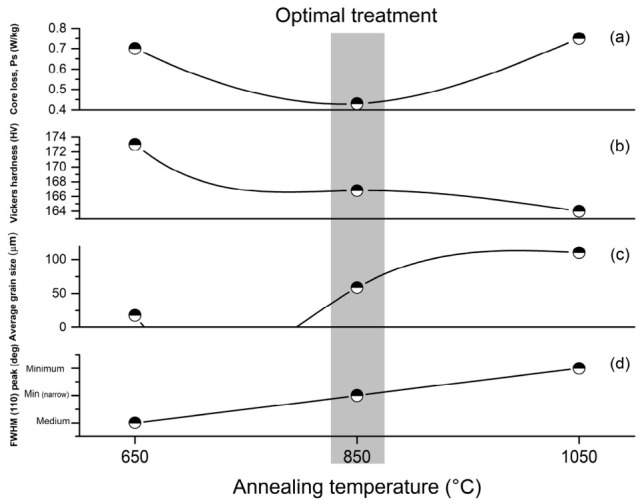
Multi-variable correlation of the M4 steel toroidal cores as a function of annealing temperature: (**a**) specific core losses (W/kg), (**b**) Vickers hardness (HV), (**c**) average grain size, and (**d**) XRD (110) peak FWHM. The shaded region indicates the optimal processing window (850 °C), where the balance between stress relief and grain growth yields the highest magnetic performance. The half-filled circular markers represent the experimental data points and the solid lines serve as visual trend guides.

The synergistic relationship between the post-processing thermal treatment and the resulting magnetic performance is summarized in Figure 9. A clear correlation is observed between the reduction in core losses and the evolution of structural parameters. As the annealing temperature reaches the optimal point of 850 °C for 90 min, the values drop to their minimum (0.43 W/kg), which directly corresponds to the nadir of Vickers hardness (166.8 HV).

This convergence suggests that the recovery and primary recrystallization mechanisms effectively minimized the internal stresses and dislocation density, as further evidenced by the narrowing of the XRD (110) peak (FWHM). Furthermore, the increase in average grain size to approximately 59 µm at 850 °C facilitates domain wall motion without entering the regime of anomalous losses observed at higher temperatures (1050 °C). This multi-variable alignment validates the factorial optimization and confirms that the structural relaxation of the toroidal cores is the primary driver for the 49% improvement in energy efficiency compared to the as-received wound cores state.

## 4. Conclusions

The present study successfully optimized the secondary annealing parameters for M4 grain-oriented electrical steel (GOES) toroidal cores through a 3^2^ full-factorial experimental design. The results provide clear evidence of the restoration of magnetic properties following the mechanical degradation introduced by industrial slitting and winding operations. The experimental data identified that an annealing temperature of 850 °C with a soaking time of 90 min is the optimal condition, yielding the minimum specific core loss of 0.43 W/kg, which represents a 49% reduction from the as-received wound cores value of 0.85 W/kg. Extending the soaking time to 120 min at the same temperature still maintains improved performance relative to the as-received wound cores state—achieving a 32% loss reduction to 0.58 W/kg—but the slight increase with respect to the 90 min condition indicates the onset of incipient grain growth effects that progressively counteract the benefits of stress relief.

The primary contribution of this work is the validation of the secondary annealing treatment applied directly to fully assembled toroidal cores under vacuum atmosphere, a distinguishing feature with respect to conventional stress-relief practices performed on individual laminations. This approach effectively eliminates the residual internal stresses and increased dislocation density induced by industrial slitting and winding, which are responsible for domain wall pinning and the consequent increase in magnetic losses. Microstructural analysis confirmed that the optimized annealing cycle promotes complete primary recrystallization and dislocation annihilation, as evidenced by the reduction in average Vickers hardness from 176.6 HV to 166.8 HV and the reinforcement of the Goss texture ({110} <001>) confirmed by XRD. Critically, these microstructural improvements were achieved without compromising the integrity of the Carlite insulating coating, which retained a stable thickness of 1.67–1.83 µm after the optimized treatment. EDS and ICP-OES analyses further verified the chemical stability of the substrate, while the beneficial decarburization—carbon reduced from 0.013 wt.% to below the detection limit—contributed additionally to hysteresis loss reduction by eliminating interstitial carbon atoms as domain wall pinning centers.

Finally, a critical upper temperature limit was identified at 1050 °C, at which specific core losses increased to 0.75 W/kg despite a continued decrease in hardness. This apparent decoupling between mechanical softening and magnetic performance confirms that excessive grain growth and the associated degradation of Goss texture sharpness—rather than residual stress alone—govern core loss behavior at elevated annealing temperatures. These findings have direct industrial relevance, demonstrating that precise control of the annealing cycle is essential for manufacturers of high-precision current transformers operating under international energy efficiency standards. The optimized parameters provide a validated industrial protocol for recovering magnetic efficiency in wound toroidal cores without compromising coating integrity. Future research will focus on evaluating the long-term magnetic stability of these optimized cores under high-frequency operating conditions and on exploring the influence of alternative insulating coatings to further extend the applicable temperature window for stress-relief annealing.

## Figures and Tables

**Table 1:** Specified maximum specific core losses (W/kg) for GOES grades M4, M5, and M6 evaluated at magnetic flux densities of 1.5 T and 1.7 T (50 Hz).

Type	1.5 Tesla (W/kg)	1.7 Tesla (W/kg)
M4 (Fe M 89-27)	0.85	1.25
M5 (Fe M 97-30)	0.9	1.3
M6 (Fe M 111-35)	1	1.5

**Table 2:** Full-factorial 3^2^ experimental run matrix for secondary annealing optimization of M4 grain-oriented toroidal cores.

Treatment	Temperature (°C)	Time (min)
Run 1	650	60
Run 2	650	90
Run 3	650	120
Run 4	850	60
Run 5	850	90
Run 6	850	120
Run 7	1050	60
Run 8	1050	90
Run 9	1050	120
Run 1R	650	60
Run 2R	650	90
Run 3R	650	120
Run 4R	850	60
Run 5R	850	90
Run 6R	850	120
Run 7R	1050	60
Run 8R	1050	90
Run 9R	1050	120

**Table 3:** Summary of specific magnetic core losses (W/kg) measured by the Epstein frame method (IEC 60404-2, 1.5 T, 50 Hz) for all 18 experimental runs of the 3^2^ factorial design.

Run	Quantity	Batch A7177	Batch A7178	Temperature (°C)	Time (min)	Replicate	Core Losses (W/kg)
Run 1	400	200	200	650	60	1	0.65
Run 1R	400	200	200	650	60	2	0.65
Run 2	400	200	200	650	90	1	0.66
Run 2R	400	200	200	650	90	2	0.66
Run 3	400	200	200	650	120	1	0.67
Run 3R	400	200	200	650	120	2	0.67
Run 4	400	200	200	850	60	1	0.52
Run 4R	400	200	200	850	60	2	0.52
Run **5**	**400**	**200**	**200**	**850**	**90**	**1**	**0.43**
Run **5R**	**400**	**200**	**200**	**850**	**90**	**2**	**0.43**
Run 6	400	200	200	850	120	1	0.58
Run 6R	400	200	200	850	120	2	0.58
Run 7	400	200	200	1050	60	1	0.7
Run 7R	400	200	200	1050	60	2	0.7
Run 8	400	200	200	1050	90	1	0.72
Run 8R	400	200	200	1050	90	2	0.72
Run 9	400	200	200	1050	120	1	0.75
Run 9R	400	200	200	1050	120	2	0.75

**Table 4:** Rockwell HR15T superficial hardness and converted Vickers hardness (HV) values for the as-received wound cores and the optimally annealed condition (850 °C, 90 min), illustrating the hardness reduction associated with recovery and primary recrystallization.

Type	HR15T	HV
As received	86.4 ± 0.8	176.6 ± 0.6
Annealed	84.9 ± 1.2	166.8 ± 0.9

**Table 5:** Bulk chemical composition (wt.%) of M4 steel in heat-treated and as-received conditions, determined by ICP-OES (for major elements) and CHNS-O elemental analysis (for carbon).

Type	Al	Cr	Cu	Mn	Ni	Si	Ti	P	S	Zn	Mg	C	Fe
Annealed-850 °C	0.027	0.039	0.004	0.093	0.006	3.816	0.008	0.028	0.002	0.001	0.067	ND	Balance
As received	0.024	0.041	0.004	0.091	0.006	3.759	0.008	0.016	0.003	0.001	0.067	0.013	Balance

## Data Availability

The original contributions presented in the study are included in the article, further inquiries can be directed to the corresponding authors.

## Abbreviations

The following abbreviations are used in this manuscript:

BCC	Body-centered cubic structure
EDS	Energy dispersive spectroscopy
GOES	Grain-oriented electrical steel
HV	Vickers hardness
ICP-OES	Inductively coupled plasma optical emission spectroscopy
SEM	Scanning electron microscopy
XRD	X-ray diffraction

## References

[1] Rodriguez-Sotelo D., Rodriguez-Licea M.A., Araujo-Vargas I., Prado-Olivarez J., Barranco-Gutiérrez A.-I., Perez-Pinal F.J. (2022). Power losses models for magnetic cores: A review. Micromachines.

[2] Serway R.A., Jewett J.W. (2008). Física Para Ciencias e Ingeniería.

[3] Comisión Federal de Electricidad CFE (2022). Transformadores y Autotransformadores de Potencia de 10 MVA y Mayores, Especificación CFE k0000-06. Mexico. https://lapem.cfe.gob.mx/normas/pdfs/x/K0000-06.pdf.

[4] Fatla O.M.H., Robinson F.C.J., Jweeg M.J., Beynon N., Valera-Medina A., Aljibori H.S.S., Mohammed M.N., Abdullah O.I. (2024). Technologies for high-temperature batch annealing of grain-oriented electrical steel: An overview. Open Eng..

[5] Hotaka H., Hutchinson B., Kubota T. (2003). The production mechanism of extremely sharp Goss orientation development in HI-B material. J. Magn. Magn. Mater..

[6] Sakakura A., Wada T., Matsumoto F., Ueno K., Takashima K., Kawashima M. (1975). Recent developments in magnetic properties of grain-oriented silicon steel with high permeability. AIP.

[7] Pluta W.A., Moses A.J. (2018). Prediction of angular variation of specific total loss of Goss-oriented electrical steel. Phys. B Condens. Matter.

[8] Tian X., Kuang S., Li J., Guo J., Feng Y. (2021). Investigation of Primary Recrystallization and Decarbonization with Different Heating Rates of Intermediate Annealing Using Nb-Containing Grain-Oriented Silicon Steel. Metals.

[9] Littmann M.F. (1967). Structures and Magnetic Properties of Grain-Oriented 3.2% Silicon—Iron. J. Appl. Phys..

[10] Li Z., Li Y., Qin Y., Hu X., Liu X., Pei R., Zeng L. (2025). Magnetic anisotropy investigation of grain-oriented electrical steels for a wide range of temperatures. AIP Adv..

[11] Fu Y.J., Jiang Q.W., Wang B.C., Yang P., Jin W.X. (2013). Morphology and Influencing Factors of Forsterite Film in Grain-Oriented Silicon Steel. J. Iron Steel Res. Int..

[12] Liang Y.F., Ye F., Lin J.P., Wang Y.L., Chen G.L. (2010). Effect of annealing temperature on magnetic properties of cold-rolled high-silicon steel thin sheet. J. Alloys Compd..

[13] Comisión Federal de Electricidad (CFE) (2006). Centros de Control de Motores de Baja Tensión de Corriente Alterna, Especificación CFE V6300-21; Mexico. https://lapem.cfe.gob.mx/normas/carga_pagina.asp?pag=V6300-21.pdf.

[14] Kubota T., Fujikura M., Ushigami Y. (2000). Recent progress and future trend on grain-oriented silicon steel. J. Magn. Magn. Mater..

[15] Fu Y., Fan L. (2025). Microstructure and Texture Evolution of High Permeability Grain-Oriented Silicon Steel. Metals.

[16] Hasirci C., Karaagac O., Köçkar H. (2019). Superparamagnetic zinc ferrite: A correlation between high magnetizations and nanoparticle sizes as a function of reaction time via hydrothermal process. J. Magn. Magn. Mater..

[17] Szumiata T., Rekas P., Gzik-Szumiata M., Nowicki M., Szewczyk R. (2024). The Two-Domain Model Utilizing the Effective Pinning Energy for Modeling the Strain-Dependent Magnetic Permeability in Anisotropic Grain-Oriented Electrical Steels. Materials.

[18] Hebri M.A., Bauw G., Lecointe J.-P., Duchesne S., Fawaz S., Zito G., Arslane I., Abdelli A., Maier A., Vandeginen C. (2025). Non-oriented electrical steel under bending and annealing effects. Arch. Electr. Eng..

[19] Hernandez I., Olivares-Galvan J.C., Georgilakis P.S., Canedo J.M. (2010). A Novel Octagonal Wound Core for Distribution Transformers Validated by Electromagnetic Field Analysis and Comparison with Conventional Wound Core. IEEE Trans. Magn..

[20] Cheng L., Ma G., Chen X., Yang F., Meng L., Yang Y., Li G., Dong H. (2020). Evolutions of microstructure and magnetic properties of heatproof domain-refined silicon steel during an-nealing and its application. J. Magn. Magn. Mater..

[21] Toktaş A., Çelik N. (2025). Recovering of deteriorated iron loss parameter of sheared and perforated grain-oriented electrical steels. Ironmak. Steelmak..

[22] Stasac C.-O., Tomșe A.-D., Arion M.-N., Bandici L., Hathazi F.-I. (2024). Effect of Heat-Treatment Process on Magnetic Characteristics of Grain-Oriented Electrical Steel. Processes.

[23] Hutchinson B., Wang F., Lozinko A., Broddefalk A. (2024). Effects of stress relief annealing in NGO steels. J. Magn. Magn. Mater..

[24] Alves E.M.M., Silveira C.C., Júnior J.R.d.O., Landgraf F.J.G. (2024). Magnetic anisotropy reduction of non-grain oriented electrical steel due to different stress relief annealing temperatures. J. Magn. Magn. Mater..

[25] Gao Q., Wang X., Li J., Cao L., Gong J., Li B. (2024). Effect of Gradient Heat Conduction on Secondary Recrystallization of Grain-Oriented Silicon Steel. Metals.

[26] Chu S., Xiang L., Guo F., Qiu S. (2025). Effect of Annealing Temperature on Microstructure, Texture, and Magnetic Properties of Non-Oriented Silicon Steel for Electric Vehicle Traction Motors. Metals.

[27] Miyagi D., Miki K., Nakano M., Takahashi N. (2010). Influence of Compressive Stress on Magnetic Properties of Laminated Electrical Steel Sheets. IEEE Trans. Magn..

[28] Cheng L., Han Y., Ma G., Wang T. (2025). Quantitative relationship between microstructure, grain orientation, and magnetic properties for grain-oriented electrical steels. J. Magn. Magn. Mater..

[29] Kang S., Lee S. (2025). Optimizing the Manufacturing Process Control of Si-Based Soft Magnetic Composites. Materials.

[30] Niu C., Zhang N., Tu Y., Meng L., Yang Y. (2025). Effect of Tensile Stress Annealing on the Texture. Grain Size and Magnetic Properties of Ultra-Thin Grain-Oriented Silicon Steel. Materials.

[31] Liang R., Sun C., Li Q. (2023). Evolution of Microstructure and Texture in Grain-Oriented 6.5% Si Steel Processed by Rolling with Intrinsic Inhibitors and Additional Inhibitors. Materials.

[32] (2008). Magnetic Materials—Part 2: Methods of Measurement of the Magnetic Properties of Electrical Steel Strip and Sheet by Means of an Epstein Frame.

[33] Arlazarov A., Gouné M., Bouaziz O., Hazotte A., Petitgand G., Barges P. (2012). Evolution of microstructure and mechanical properties of medium Mn steels during double annealing. Mater. Sci. Eng. A.

[34] (2026). Standard Specification for Flat-Rolled, Grain-Oriented, Silicon-Iron, Electrical Steel, Fully Processed Types.

[35] (2022). Methods of Measurement of the Magnetic Properties of Electrical Steel Sheet and Strip by Means of a Single Sheet Tester.

[36] (2018). Standard Classification of Insulating Coatings for Electrical Steels by Composition, Relative Insulating Ability and Application.

[37] Cleveland-Cliffs (2023). Grain-Oriented Electrical Steels (GOES), Lite Carlite and Mill-Anneal, Product Data Sheet. https://d1io3yog0oux5.cloudfront.net/_da2924d88f307639ddbdfeb816787701/clevelandcliffs/db/1190/10493/file/CLF_ProductData_LiteCarliteAndMillAnneal_022023.pdf.

[38] Dawood K., Cınar M.A., Alboyacı B., Sonmez O. (2017). Efficient finite element modeling for the calculation of transformer no-load losses. Int. J. Eng. Appl. Sci..

[39] Dawood K., Tursun S. (2024). Numerical and experimental comparison of the no load losses in the different grain oriented electrical steel materials. e-Prime-Adv. Electr. Eng. Electron. Energy.

[40] Mago M.G., Valles L., Olaya J.J., Espejo E., Arango P., Sierra M. (2018). Failure analysis in silicon steel sheet in distribution transformers. Effect of the zone of precedence of the transformers. Rev. ION.

[41] Sahu S., Rao B.K., Sahoo S.K., Jammalamadaka S.N., Basheed G.A. (2026). Influence of post-annealing on magnetic anisotropy, magnetic domain-state configuration and broadband fer-romagnetic resonance in Co60Fe20B20 thin films. J. Magn. Magn. Mater..

[42] Petryshynets I., Kováč F., Füzer J., Falat L., Puchý V., Kollár P. (2019). Evolution of Power Losses in Bending Rolled Fully Finished NO Electrical Steel Treated under Uncon-ventional Annealing Conditions. Materials.

[43] Normas Oficiales Mexicanas (NOM) (2010). Requisitos de Seguridad y Eficiencia Energética Para Transformadores de Distribución.

[44] Dias M., Landgraf F. (2020). Compressive stress effects on magnetic properties of uncoated grain-oriented electrical steel. J. Magn. Magn. Mater..

[45] Boggavarapu S.R., Baghel A.P.S., Chwastek K., Kulkarni S.V., Daniel L., de Campos M.F., Nlebedim I.C. (2025). Modelling of angular behavior of core loss in grain-oriented laminations using the loss separation approach. J. Supercond. Nov. Magn..

[46] De Araújo J.R.L., Silva H.F., Reis A.B.C.D., Bitencourt M. (2019). Effect of primary and secondary annealing at grain-oriented electrical steel. Res. Soc. Dev..

[47] Taguchi S., Yamamoto T., Sakakura A. (1974). New grain-oriented silicon steel with high permeability Orientcore H-B. IEEE Trans. Magn..

[48] Belgrand T., Lemaître R., Benabou A., Blaszkowski J., Wang C. (2018). Thin grain oriented electrical steel for PWM voltages fed magnetic cores. AIP Adv..

[49] Wan Y., Chen W., Zhao Q. (2016). Effect of Ag Content on the Microstructure and Magnetic Properties of Grain-oriented Silicon Steels. ISIJ Int..

[50] Di Cunto J.P., Valls Martin R., Gomes Landgraf F.J. (2022). Magnetic Anisotropy and Loss Separation in Grain Oriented Electrical Steels (Goes). SSRN Electron. J..

[51] Qin J., Yang P., Mao W., Ye F. (2015). Effect of texture and grain size on the magnetic flux density and core loss of cold-rolled high silicon steel sheets. J. Magn. Magn. Mater..

[52] Price K., Goode B., Power D. (2016). Grain-oriented electrical steels for power and distribution transformers. Ironmak. Steelmak..

[53] Jahangiri M., Bayani H., Ardestani M., Mehdizadeh M. (2022). Core loss reduction in grain oriented silicon steel sheets by two sided laser scribing in the presence of a magnetic field. J. Alloys Compd..

[54] (2024). Standard Test Methods for Determining Average Grain Size.

[55] Leuning N., Heller M., Jaeger M., Korte-Kerzel S., Hameyer K. (2023). A new approach to measure fundamental microstructural influences on the magnetic properties of electrical steel using a miniaturized single sheet tester. J. Magn. Magn. Mater..

[56] Mehdi M., He Y., Hilinski E.J., Kar N.C., Edrisy A. (2019). Non oriented electrical steel with core losses comparable to grain oriented electrical steel. J. Magn. Magn. Mater..

[57] Mörée G., Leijon M. (2024). Iron loss models: A review of simplified. models of magnetization losses in electrical machines. J. Magn. Magn. Mater..

[58] You J., Yu H., Liang H., Xie Y., Ding D. (2022). A multi-parameter model of heat treatment process for soft magnetic materials on performance of HSERs. Chin. J. Aeronaut..

[59] Ouyang G., Chen X., Liang Y., Macziewski C., Cui J. (2019). Review of Fe 6.5%Si high silicon steel—A promising soft magnetic material for sub kHz application. J. Magn. Magn. Mater..

[60] Roppert K., Kaltenbacher M., Domenig L., Daniel L. (2025). Magnetoelastic Vector Hysteresis Modeling for Electromagnetic Devices: A Combination of a Multiscale Model with the Energy-Based Hysteresis Framework. IEEE Trans. Magn..

[61] Paltanea G., (Paltanea) V.M., Antoniac A., Nemoianu I.V., Gavrila H. (2024). Mechanical and Magnetic Properties Variation in Non-Oriented Electrical Steels with Different Cutting Technology. Materials.

[62] Kronmüller H., Hertel R. (2000). Computational micromagnetism of magnetic structures and magnetisation processes in small particles. J. Magn. Magn. Mater..

[63] Valchev V., Bossche A.V.D., Sergeant P. (2008). Core losses in nanocrystalline soft magnetic materials under square voltage waveforms. J. Magn. Magn. Mater..

[64] Boehm L., Hartmann C., Gilch I., Stoecker A., Kawalla R., Wei X., Hirt G., Heller M., Korte-Kerzel S., Leuning N. (2021). Grain size influence on the magnetic property deterioration of blanked non-oriented electrical steels. Materials.

[65] (2019). Standard Test Method for Alternating-Current Magnetic Properties of Materials at Power Frequencies Using Wattmeter-Ammeter-Voltmeter Method and 25-cm Epstein Test Frame.

[66] Tiismus H., Kallaste A., Belahcen A., Tarraste M., Vaimann T., Rassõlkin A., Asad B., Ghahfarokhi P.S. (2021). AC Magnetic Loss Reduction of SLM Processed Fe-Si for Additive Manufacturing of Electrical Machines. Energies.

[67] Stoyka V., Kováč F., Stupakov O., Petryshynets I. (2010). Texture evolution in Fe–3% Si steel treated under unconventional annealing conditions. Mater. Charact..

[68] Daniels B., Overboom T.T., Lomonova E.A. (2020). Coupled statistical and dynamic loss prediction of high-permeability grain-oriented electrical steel. Eur. Phys. J. Appl. Phys..

[69] Daniels B., Overboom T., Lomonova E. Hysteresis and Loss Prediction for High-Permeability Grain-Oriented Electrical Steel by Material Characterization. Proceedings of the 2019 19th International Symposium on Electromagnetic Fields in Mechatronics, Electrical and Electronic Engineering (ISEF).

[70] Weidenfeller B., Riehemann W. (2005). Effects of surface treatments on the hysteresis losses of GO iron silicon steel. J. Magn. Magn. Mater..

[71] Tiismus H., Kallaste A., Belahcen A., Vaimann T., Rassõlkin A., Lukichev D. (2020). Hysteresis Measurements and Numerical Losses Segregation of Additively Manufactured Silicon Steel for 3D Printing Electrical Machines. Appl. Sci..

